# Clinical and Microbiological Evaluation of 0.2% Tea Tree Oil Mouthwash in Prevention of Dental Biofilm-Induced Gingivitis

**DOI:** 10.3390/dj13040149

**Published:** 2025-03-28

**Authors:** Adarsha Mahapatra, Saurav Panda, Margherita Tumedei, Sital Panda, Abhaya Chandra Das, Manoj Kumar, Massimo Del Fabbro

**Affiliations:** 1 Institute of Dental Sciences, Siksha ‘O’ Anusandhan University, Bhubaneswar 751030, India; mahapatraadarsha452@gmail.com (A.M.); dr.sitalpanda@gmail.com (S.P.); 2 Department of Periodontics, Institute of Dental Sciences, Siksha ‘O’ Anusandhan University, Bhubaneswar 751030, India; sauravpanda@soa.ac.in (S.P.); abhayadas@soa.ac.in (A.C.D.); manojkumar@soa.ac.in (M.K.); 3 Department of Biomedical, Surgical and Dental Sciences, Università degli Studi di Milano, 20122 Milan, Italy; massimo.delfabbro@unimi.it; 4 UOC Maxillofacial Surgery and Dentistry, Fondazione IRCCS Ca’Granda Ospedale Maggiore Policlinico, 20122 Milan, Italy

**Keywords:** tea tree oil, chlorhexidine, mouthwash, antimicrobial, gingivitis

## Abstract

**Background:** Dental biofilm-induced gingivitis is a prevalent condition caused by dental plaque accumulation. Chlorhexidine mouthwash is a gold standard for plaque control but is associated with adverse effects such as tooth staining and altered taste. This study aimed to evaluate the clinical and antimicrobial effectiveness of 0.2% tea tree oil mouthwash as a natural alternative to 0.2% chlorhexidine mouthwash. **Methods:** A comparative study was conducted on 60 participants aged 18–60 years, divided into two groups: Group T (tea tree oil) and Group C (chlorhexidine), each comprising 30 participants. Clinical outcomes assessed included Plaque Index (PI), Gingival Index (GI), Bleeding on Probing (BOP), and microbiological Colony Forming Units (CFUs). Parameters were recorded at baseline, 7 days, and 28 days. **Results:** Group T exhibited significantly lower PI and BOP scores at 7 and 28 days compared to Group C (*p* < 0.05). Both groups showed comparable reductions in CFU counts, indicating similar antimicrobial efficacy. Importantly, tea tree oil had fewer adverse effects, with no reports of tooth staining or altered taste, unlike chlorhexidine. **Conclusion:** Tea tree oil mouthwash demonstrated equivalent or superior clinical outcomes compared to chlorhexidine, with fewer side effects. It is a viable and well-tolerated alternative for managing plaque-induced gingivitis, supporting further research into its long-term use and efficacy.

## 1. Introduction

The oral cavity is affected by various microorganisms, leading to the development of gingivitis and periodontal-related inflammation [1,2,3,4]. Deposition of dental plaque is the main causative factor for gingivitis, which can be controlled mainly by mouthwash application [5,6]. A remarkable reduction in dental plaque accumulation is seen by using chlorhexidine mouthwash, which is a bis-biguanide [7]. However, many associated side effects are visible, which triggered the search for alternative means.

In recent times, alternative natural remedies are preferred over chlorhexidine mouthwash. These natural substitutes have been adapted more widely due to their fewer side effects [8,9]. Many plant extracts are studied extensively in vitro for a better understanding of their therapeutic properties. Natural remedies mainly consist of essential oils that have strong antiseptic and anti-inflammatory effects, which regular studies can establish.

Nowadays, an essential oil named tea tree oil is frequently used in various medical fields such as dentistry, dermatology, etc. Tea tree oil is recognized as one of the most well-known essential oils with broad-spectrum antimicrobial activity, derived from a plant of the family Myrtaceae called *Melaleuca alternifolia* [10]. These plants are most commonly seen in Australia as arboreal species. The oil has a nutmeg-like smell and a light-yellow pine needle-like colour. It is mainly composed of two components like terpineol-4 and 1,8-cineole. The percentage composition of terpineol-4 should be higher than 30%, and cineole should be less than 15%. At a higher percentage, cineole constitutes a toxic and irritating component of the oil, so a proper ratio should be maintained.

Various studies have aimed to evaluate the improvement in clinical outcomes and the antiplaque effect [11,12]. A study was performed by Ripari et al. in 2020 [11] where 0.12% chlorhexidine mouthwash and 0.2% tea tree oil as a mouthwash was administered to participants aged 18–60 years for 14 days and showed no significant difference in antiplaque effect. Rahman et al. in 2014 [10] performed a similar clinical evaluation in young adults, 22.55 ± 1.79 years old, to find a similar effect. Kamath in 2020 [13] compared the clinical efficacy of tea tree oil and 0.2% chlorhexidine mouthwash in paediatric patients of 8–12 years old to prove the beneficial effect of tea tree oil over the gold standard. Bharadwaj et al. 2021 [14] conducted a randomized clinical trial on young adults and obtained a significant improvement in clinical outcomes evaluated using 0.2% tea tree oil over 0.2% chlorhexidine mouthwash.

However, none of them evaluated the antimicrobial effect, which could indicate the reduction in the periodontopathogens involved in dysbiosis. Therefore, the aim of our study is not only to evaluate the impact of tea tree oil clinically but also to test the antimicrobial efficacy of the same compared to 0.2% chlorhexidine mouthwash, which remains the first line of antimicrobial treatment despite many side effects. This study’s main aim and objective was to compare the clinical and antimicrobial effectiveness of 0.2% tea tree oil with 0.2% chlorhexidine mouthwash for the management of dental biofilm-induced gingivitis.

## 2. Materials and Methods

This study was conducted with 60 participants within the age group of 18–60 years, who reported to the Out-patient Department of Periodontics, Institute of Dental Sciences, Bhubaneswar. This study was approved by the Institutional Review Board and cleared the ethical approval from IEC, Institute of Dental Sciences, Bhubaneswar with registration number (Approval date: 10 October 2023 IEC-IDS/IDS/SOA/2023/I-28). This study was conducted over a total duration of 2 months between January and March 2024.

### 2.1. Preparation of 0.2% Tea Tree Oil Mouthwash

The tea tree oil extract was procured from Mother Herbs Private Ltd., New Delhi, India. The extract was authenticated by the Department of Pharmacology, Siksha O Anusandhan University, Bhubaneswar. The mouthwash was prepared in a sterile beaker by mixing 2 g of tween 80 and 2 mL of pure tea tree oil; then, the mixture was added to two Liters of distilled water to make up the required concentration of 0.2% [15].

### 2.2. Sample Size Estimation

The estimated sample size calculation was carried out using the Reddy et al. 2020 [15] pilot study. A sample size of 24 participants per group was calculated under 80% power to achieve a significance of 0.5 difference in total plaque index between both groups. Considering attrition, a total of 30 patients were included in this study.

### 2.3. Inclusion and Exclusion Criteria

Healthy participants with mild to moderate gingivitis, 18–35 years of age, having all their teeth except their third molars, and presenting no allergies, were included. Those with smoking habits; presenting with caries, periodontitis, or other oral disease/condition; with use of any antibiotic therapy in the previous 6 months; with use of antimicrobial agents two weeks before recruitment; using orthodontic or prosthodontic appliances; with immuno-compromised conditions; or who were pregnant or lactating were excluded from this study.

### 2.4. Randomization

A total of 60 participants were involved, with 30 participants randomly assigned to each group using a coin-toss method. Participants who received 0.2% tea tree oil mouthwash for 7 days were included in Group T, and participants who received 0.2% chlorhexidine mouthwash (Hexidine Mouthwash, ICPA Health Products, Mumbai, India) for 7 days were included in Group C.

### 2.5. Outcomes Assessed

The following primary outcomes were assessed at Baseline, 7 days, and 28 days of the intervention:

- Gingival Index (GI)—Löe and Sillness index.
- Plaque Index (PI)—Sillness and Löe index.
- Percentage of Bleeding on Probing sites (% BOP).
- Microbiological Assessment—Total Colony Count (CFU—Colony Forming Unit).

Subjective and objective patient perceptions of taste, burning sensation, and dryness along with ulcer formation, staining of the teeth, staining of the tongue, and allergy were also recorded as a secondary outcome of this study.

### 2.6. Procedure

All participants underwent thorough scaling and root planing (SRP) upon recruitment, performed by an expert periodontist to ensure a plaque-free baseline environment. They were advised to refrain from brushing for the following 7 days to allow for plaque build-up and the initiation of experimental gingivitis.

Day 0 (Baseline): Participants of both groups T and C were instructed to brush their teeth twice daily using the modified Bass brushing technique without toothpaste, followed by using the provided mouthwash twice daily for the next 7 days. Both groups received the mouthwash in concealed white bottles without labels. Baseline parameters were recorded, and unstimulated saliva samples were collected in Eppendorf tubes during morning hours.

Week 1 (Day 7): The participants were asked to stop using both mouthwashes and return to their usual brushing habits twice daily. Parameters at 1 week were recorded, and unstimulated saliva samples were collected in Eppendorf tubes during morning hours.

Week 4 (Day 28): Parameters at 4 weeks were recorded, and unstimulated saliva samples were collected in Eppendorf tubes during morning hours.

The unstimulated salivary samples were collected from all the participants before brushing at all follow-ups. Each sample was immediately subjected to sonication (Sonicator 500 W, Sigma Aldrich, St. Louis, MO, USA) at 5% amplitude, with a 9.9-s cycle and six pulses. The samples were then diluted in saline solution at 1:100 and 1:1000 ratios. A 5 µL aliquot of each diluted sample was spread onto small Petri dishes (5 × 2 cm) containing blood agar (agar base supplemented with 5% sheep blood). The blood agar plates (HIMEDIA Co., Maharashtra, India) were incubated in an aerobic incubator at 37 °C for 48 h.

The total colony counts on the Petri dishes were assessed using a manual colony counter under a microscope. The evaluation process involved visual identification and quantifying discrete bacterial colonies that had developed on the blood agar surface.

#### Primary and Secondary Outcomes

The primary outcome was to assess the change in the recorded clinical parameters and bacterial CFUs formed after 7 days and 28 days from the baseline.

The secondary outcome was to evaluate the patients’ acceptance of the mouthwash and the tolerability of the side effects. A patients satisfaction survey was performed wherein questions were asked on altered taste sensation, burning sensation, and staining of teeth. The answers were recorded as “Yes” if present, and “No” if absent.

### 2.7. Statistical Analysis

The statistical analysis for this study was performed using SPSS 20.0 software. Normality tests were conducted to assess whether the data followed a normal distribution using the Kolmogorov–Smirnov test and Shapiro–Wilk test. For normally distributed data, independent *t*-tests were used to compare the means of continuous variables such as PI, GI, BOP, and CFUs between Group C and Group T. The results were presented as mean values with standard deviations and standard error of the mean. For each comparison, a *p*-value of less than 0.05 was considered statistically significant.

## 3. Results

A total of 60 participants were enrolled in this study, with 30 participants in each group. The average age was 34.7 years (standard deviation: 3.2 years). Of these, 45% were male, and 55% were female. The groups were comparable in terms of age, gender, and baseline clinical indices.

## 4. Clinical Outcomes

### 4.1. Plaque Index (PI)

The mean PI at baseline was similar for both Group C (chlorhexidine) and Group T (tea tree oil), with no significant difference (*p* = 0.496). However, after 7 days, PI was significantly lower in Group T (0.8470 ± 0.15370) compared to Group C (1.0290 ± 0.10847), with a *p*-value of 0.003. At 28 days, Group T also demonstrated a significantly lower PI (0.6020 ± 0.08942) compared to Group C (0.8410 ± 0.19428), with a *p*-value of 0.001 (Table 1).

### 4.2. Gingival Index (GI)

The GI at baseline and after 7 days showed no significant difference between the two groups (*p* > 0.05). At 28 days, Group T had a slightly higher GI (0.3489 ± 0.0757) than Group C (0.2456 ± 0.234), but this difference was not statistically significant (*p* = 0.387) (Table 1).

### 4.3. Bleeding on Probing (BOP)

Baseline BOP levels were not significantly different between Group C (33.1330 ± 4.05271) and Group T (35.3430 ± 2.91682), with a *p*-value of 0.089. After 7 days, there was still no significant difference (*p* = 0.270). However, at 28 days, Group T exhibited a significantly lower BOP (18.7810 ± 1.34418) compared to Group C (26.8650 ± 4.80070), with a *p*-value of 0.004 (Table 1).

### 4.4. Microbiological Outcomes

CFUs were measured at baseline, 7 days, and 28 days. At baseline, both groups had similar CFU counts, with no significant difference (*p* = 0.433). At 7 days, the difference between the groups was also not significant (*p* = 0.294). At 28 days, both groups experienced a reduction in CFUs, with no significant difference (*p* = 0.329) (Table 1).

#### Subjective and Objective Evaluations

Regarding subjective experiences, 24 out of 30 participants in Group T found the taste acceptable, compared to 21 in Group C. Both groups had six participants experiencing a burning sensation, but none reported dryness or soreness. No ulcer formation or allergic reactions were observed in either group (Table 2).

Tooth staining was reported in Group C, with 3 out of 30 participants experiencing it, while none in Group T reported such issues (Table 2).

## 5. Discussion

This study aimed to evaluate the clinical and antimicrobial effectiveness of 0.2% tea tree oil mouthwash compared to 0.2% chlorhexidine in managing plaque-induced gingivitis. The findings indicate that tea tree oil mouthwash offers significant antiplaque and anti-inflammatory benefits while exhibiting fewer side effects than chlorhexidine. The implications of these findings are substantial, as they contribute to the growing body of evidence supporting the use of natural alternatives in oral healthcare.

Tea tree oil’s antiplaque and antimicrobial properties can be attributed to its high content of terpinene-4-ol, the primary active component, known for its broad-spectrum antimicrobial activity. The oil is derived from Melaleuca alternifolia, a tree native to Australia, and has been used in traditional medicine for its antiseptic and anti-inflammatory effects [16]. Terpinene-4-ol disrupts bacterial cell membranes, leading to cell death, and exhibits anti-inflammatory effects by inhibiting pro-inflammatory molecules production [17]. This dual action is particularly beneficial in treating gingivitis, where both microbial colonization and inflammation are key factors. While chlorhexidine is considered the gold standard [18] in mouthwash for its strong antibacterial properties, it has several well-documented side effects, including tooth staining, altered taste, and mucosal irritation [19]. In contrast, tea tree oil presents a more natural alternative with fewer adverse effects, as shown in this study, where participants in the tea tree oil group reported higher taste acceptability and no instances of tooth staining or ulcer formation. This distinction makes tea tree oil an attractive option for individuals seeking a gentler yet effective mouthwash.

Our study demonstrated that tea tree oil significantly reduced the Plaque Index and Bleeding on Probing after seven days of use, with further reductions observed at 28 days. These results suggest that tea tree oil exerts a robust antiplaque effect comparable to chlorhexidine. The reduction in plaque accumulation can be attributed to tea tree oil’s ability to disrupt bacterial biofilms, a key factor in plaque formation and gingival inflammation [20]. The Gingival Index showed no statistically significant difference between the two groups, indicating that both treatments effectively reduced gingival inflammation. This suggests that tea tree oil’s anti-inflammatory properties are on par with those of chlorhexidine [21]. Our results are in accordance with a finding of a systematic review [22], where tea tree oil is found to be superior to chlorhexidine in reducing signs of gingival inflammation; however, chlorhexidine is superior to tea tree oil in inhibiting plaque formation, probably due to its increased substantivity; therefore, it may be used as an alternative to chlorhexidine for reduction of gingival inflammation in conjunction with efficient plaque control measures.

The ability of tea tree oil to mitigate gingival inflammation without causing additional side effects, such as mucosal irritation, underscores its potential as a safe and effective treatment for plaque-induced gingivitis [23]. The microbiological assessment revealed a decrease in CFUs in both groups, with no significant difference between them. This suggests that tea tree oil exhibits antimicrobial properties comparable to chlorhexidine. The broad-spectrum antimicrobial activity of tea tree oil, primarily attributed to terpinene-4-ol, contributes to its ability to reduce bacterial load in the oral cavity by disrupting bacterial cell membranes and inhibiting pro-inflammatory cytokines [14,20].

Tea tree oil provides a dual-action benefit—targeting both microbial colonization and inflammatory responses, which are critical in gingivitis progression. The findings of this study align with previous research that has evaluated the efficacy of tea tree oil in oral health. Reddy et al. [15] demonstrated that a mouth rinse with 0.2% tea tree oil has a similar effect as chlorhexidine in the reduction of plaque and gingival scores. Further, Ripari et al. [11] reported no significant difference in antiplaque effects between 0.12% chlorhexidine and 0.2% tea tree oil, reinforcing the idea that tea tree oil can serve as an effective alternative to chlorhexidine in plaque control. To prove its antimicrobial activity, another study by Khalil et al. [24] showed a significant reduction in the mean number of Streptococcus mutans colonies and concluded that tea tree oil mouthwash has significant antimicrobial activity and could be utilized as a natural substitute to chlorhexidine. Similarly, Bharadwaj et al. [14] demonstrated significant clinical improvement in gingival health with tea tree oil use among young adults. These studies, together with the current findings, suggest that tea tree oil offers a promising natural alternative for individuals seeking effective plaque control without the side effects commonly associated with chlorhexidine. On the other hand, it was reported that tea tree oil may have dose-dependent cytotoxic effects in vitro, with concentrations above 0.5% affecting gingival fibroblasts and epithelial cells [25]. Conversely, no in vitro genotoxic effects of tea tree oil were demonstrated [26]. In vitro studies showed that chlorhexidine may have a cytotoxic effect on gingival fibroblasts, even at a 0.12% concentration [27,28]

Our study used unstimulated saliva as the sample, as it represents a broader aerobic microbial environment of the oral cavity. Collecting gingival crevicular fluid in adequate volumes for microbiological assessment is technically challenging. However, it would represent a sub-gingival plaque environment including both aerobic and anaerobic microbiota. Prior research [14,15,23,24] has validated saliva as a suitable medium for evaluating oral aerobic microbiota. However, future studies are required to determine the inhibition of anaerobic bacteria to establish the complete antimicrobial effects of the mouthwash.

The findings of this study can be attributed to the combined anti-inflammatory and antibacterial properties of tea tree oil. Its antimicrobial activity primarily stems from its ability to disrupt the permeability barrier of microbial cell membranes, leading to cell lysis and death [29]. Our findings also align with those of Soukoulis and Hirsch [30], who reported that using a tea tree oil gel twice daily as a dentifrice significantly reduced gingival inflammation and bleeding in cases of severe gingivitis. Similarly, other studies [31,32] have demonstrated notable improvements in gingival health due to tea tree oil’s anti-inflammatory effects. Moreover, tea tree oil may exert its anti-inflammatory action by stimulating superoxide production in human monocytes, further contributing to its role in reducing gingival inflammation [33].

Tea tree oil and chlorhexidine have been shown to exhibit inhibitory effects on matrix metalloproteinase-8 (MMP-8), a key enzyme involved in collagen degradation and periodontal tissue destruction [34,35,36]. Tea tree oil, particularly its active component terpinen-4-ol, may reduce MMP-8 activity by modulating inflammatory pathways and downregulating pro-inflammatory cytokines that stimulate its expression [17]. Similarly, chlorhexidine has been reported to suppress MMP-8 activity, potentially through its ability to interfere with MMP activation pathways, thereby limiting collagen breakdown and supporting periodontal health [37]. This dual action of antimicrobial and MMP-8 inhibition makes both tea tree oil and chlorhexidine valuable in managing periodontal inflammation and preventing tissue degradation.

### 5.1. Strengths and Limitations

The strengths of this study include its randomized design, the use of established clinical indices for evaluation, and the microbiological assessment to confirm antimicrobial efficacy. The sample size was calculated based on a prior pilot study, ensuring sufficient statistical power to detect meaningful differences between the treatment groups.

However, this study has certain limitations. The relatively small sample size and short study duration may limit the generalizability of the findings and fail to capture long-term effects or the recurrence of gingivitis. Additionally, while adherence to the mouthwash regimen was encouraged, variations in patient compliance could have influenced the results. Another limitation is the absence of a placebo control group, which might have provided further clarity on the mouthwash’s effects relative to no treatment.

### 5.2. Future Directions

To build upon the findings of this study, future research should focus on larger, multi-centre clinical trials to validate the efficacy and safety of tea tree oil mouthwash across diverse populations. Investigating different concentrations of tea tree oil and its effects on various bacterial strains could provide deeper insights into its antimicrobial mechanisms. Long-term studies assessing patient compliance, satisfaction, and the recurrence of gingivitis will be valuable in determining the sustained benefits of tea tree oil as an alternative to conventional mouthwashes.

Additionally, exploring the potential synergistic effects of tea tree oil with other natural antimicrobial agents could enhance its clinical application. Patient-centred research, including qualitative studies on acceptance, taste perception, and overall satisfaction, will further elucidate the practicality of tea tree oil mouthwash in routine oral care.

## 6. Conclusions

Overall, the tea tree oil mouthwash demonstrated similar or superior outcomes in plaque index, gingival index, and bleeding on probing compared to chlorhexidine mouthwash. It also had fewer side effects and was better tolerated by participants, indicating its potential as a safe and effective alternative for managing plaque-induced gingivitis.

Overall, this study suggests that 0.2% tea tree oil mouthwash is a viable alternative to 0.2% chlorhexidine mouthwash for managing dental biofilm-induced gingivitis. It offers comparable antiplaque and antimicrobial effects with fewer side effects such as tooth staining and altered taste. The findings support further research to establish long-term outcomes and explore additional applications for tea tree oil in oral health.

## Figures and Tables

**Table 1:** Inter-group comparison of clinical and microbiological outcomes at all time-points.

Variables	Time-Points	Group	N	Mean	Std. Deviation	Std. Error Mean	*p* Value
Plaque Index (PI)	Baseline	C	30	1.445	0.2441	0.07719	0.496
		T	30	1.444	0.17513	0.05538	
	7 days	C	30	1.029	0.10847	0.0343	0.003 *
		T	30	0.847	0.1537	0.0486	
	28 days	C	30	0.841	0.19428	0.06144	0.001 *
		T	30	0.602	0.08942	0.02828	
Gingival Index (GI)	Baseline	C	30	0.6579	0.2567	0.06786	0.782
		T	30	0.5567	0.1984	0.0992	
	7 days	C	30	0.0567	0.118	0.0678	0.678
		T	30	0.0879	0.7896	0.06723	
	28 days	C	30	0.2456	0.234	0.04354	0.387
		T	30	0.3489	0.0757	0.02389	
Percentage of Bleeding on Probing (%BOP)	Baseline	C	30	33.133	4.05271	1.28158	0.089
		T	30	35.343	2.91682	0.92238	
	7 days	C	30	30.09	4.18029	1.32192	0.27
		T	30	29.2	1.70523	0.53924	
	28 days	C	30	26.865	4.8007	1.51812	0.004 *
		T	30	18.781	1.34418	0.42507	
Colony Forming Units (CFUs) × 10^3^	Baseline	C	30	17.8	2.658	0.841	0.433
		T	30	18	2.582	0.816	
	7 days	C	30	12.4	3.627	1.147	0.294
		T	30	13.3	3.653	1.155	
	28 days	C	30	9.6	3.307	1.046	0.329
		T	30	18.781	1.34418	0.42507	

* *p* < 0.05—statistically significant.

**Table 2:** Patient satisfaction survey (percentage of patients reporting subjective and objective issues).

Outcomes		Criteria	Group T (n = 30)	Group C (n = 30)
Subjective	Taste Acceptability	Acceptable	24	21
		Unacceptable	6	9
	Burning	Present	6	6
		Absent	24	24
	Dryness/Soreness	Present	0	0
		Absent	0	0
Objective	Ulcer Formation	Present	0	0
		Absent	0	0
	Staining of Tooth	Present	0	3
		Absent	0	27
	Staining of Tongue	Present	0	0
		Absent	0	0
	Allergy	Present	0	0
		Absent	0	0

## Data Availability

Available under request to the corresponding author.
